# A Natural Enzyme GOx‐MOF Cascade Complex Killing Zoonotic Parasite *Trichinella spiralis* by Energy Utilization Limitation and Generated Free Radicals

**DOI:** 10.1155/tbed/3527187

**Published:** 2026-09-18

**Authors:** Chuang Zhang, Xiaolei Liu, Zipei Zhang, Zhili Hao, Xu Geng, Ning Jiang, Mingyuan Liu, Zhengqiang Li, Chen Li

**Affiliations:** ^1^ State Key Laboratory for Diagnosis and Treatment of Severe Zoonotic Infectious Diseases, Key Laboratory for Zoonosis Research of the Ministry of Education, Institute of Zoonosis, and College of Veterinary Medicine, Jilin University, Changchun 130062, China, jlu.edu.cn; ^2^ Key Laboratory for Molecular Enzymology and Engineering of the Ministry of Education, College of Life Sciences, Jilin University, Changchun, China, jlu.edu.cn; ^3^ School of Pharmacy, Jilin Medical University, Jilin, Jilin Province, China, jlu.edu.cn; ^4^ Affiliated Hospital of Changchun University of Chinese Medicine, Changchun, Jilin, China, ccucm.cn

**Keywords:** glucose oxidase, MOF-525(Fe), MPG(Fe), nanozyme, *Trichinella spiralis*

## Abstract

Nanozymes offer promising alternatives to natural enzymes due to enhanced stability, tunable activity, and cost efficiency, demonstrating therapeutic potential against tumors, infections, and inflammatory disorders. *Trichinella spiralis* is an important pathogen, which globally prevalent foodborne zoonotic parasite. Chemical drug is commonly used to kill *T. spiralis*, but there are risks of chemical abuse and drug resistance. Here, we engineered MPG(Fe), a cascade nanozyme formed by covalently conjugating glucose oxidase (GOx) to a metal‐organic framework‐Fe nanozyme (MOF‐525[Fe]) nanozyme via the bifunctional PEG linker. In vitro, MPG(Fe) depleted glucose through GOx‐catalyzed conversion to gluconic acid and H_2_O_2_, while MOF‐525(Fe) subsequently transformed H_2_O_2_ into cytotoxic hydroxyl radicals (•OH). This dual‐action mechanism dose‐dependently killed intestinal‐stage adult worms and newborn larvae (NBL) of *T. spiralis*. In a BALB/c mouse model of intestinal *T. spiralis* infection, high‐dose MPG(Fe) reduced the survival of intestinal adult worms to 3.53% of the control value through the synergistic actions of glucose deprivation and hydroxyl radical (•OH)‐mediated oxidative damage. Critically, MPG(Fe) exhibited no tissue toxicity in uninfected mice. Our findings establish enzyme‐mimetic nanozymes as a novel therapeutic platform against zoonotic helminthiases.

## 1. Introduction

Eosinophils, neutrophils, and monocytes (MONs) play key roles in the host resistance to parasite invasion and migration. Increased parasite killing correlates with elevated numbers of neutrophils and eosinophils, which possess surface peroxidases (PODs) with antiparasitic activity [1–3]. In fact, H_2_O_2_ production catalyzed by eosinophil POD has been cited as evidence for an eosinophil/H_2_O_2_/Cl^−^ interaction mechanism, demonstrated to kill parasitic helminths in vitro [4]. Moreover, antibody‐triggered neutrophil POD activity has been shown to kill helminths more effectively than eosinophil POD in vitro [5].

Glucose oxidase (GOx), a natural enzyme approved for use in feed additives (as listed in China’s Catalog of Feed Additive Varieties), catalyzes the oxidation of endogenous glucose by oxygen, generating H_2_O_2_ and gluconic acid in situ [6, 7]. This reaction elevates local H_2_O_2_ concentrations, thereby enhancing the POD activity [8]. POD‐dependent oxidative reactions constitute a natural and effective antihelminthic defense mechanism. However, this mechanism relies on the recruitment and activation of host immune cells, which is delayed, energetically costly, and can be subverted by parasites. Therefore, we infer that a synthetic cascade reaction simulating the GOx/POD tandem is involved. In this reaction, GOx generates H_2_O_2_ in situ using the abundant glucose in the intestinal cavity, while the class POD nanoenzymes convert this H_2_O_2_ into more cytotoxic hydroxyl radicals (•OH). This strategy recreates and scales up the host’s natural oxidative killing in an in‐cell manner and does not rely on the host’s immune status. Such systems hold significant promise for developing considerably more effective anti‐helminthic therapies [9].

Metal‐organic frameworks (MOFs) have garnered significant attention in recent years owing to their distinctive properties, including high porosity, large surface area, tunable pore size, and compatibility with polymers, biomolecules, and metal nanoparticles [10–13]. Notably, MOF‐525(Fe) (hereafter referred to as MOF‐Fe), which utilizes iron porphyrins as ligands, has been shown to exhibit high‐level catalytic activity and thus has great potential as a nanomedicine [14–16]. The coordinated Fe atoms within its porphyrin groups confer POD‐like activity, enabling the generation of H_2_O_2_, which can subsequently be converted into hydroxyl radicals (•OH) [16, 17].

In enzymatic cascades, sequentially reacting enzymes often assemble into stable complexes to achieve efficient reaction pathways. However, simply mixing two distinct enzymes typically fails to form stable conjugates, resulting in significantly lower cascade activity compared to covalently linked enzyme systems [18]. While enzymes can adsorb electrostatically onto MOF carriers, such attachments are often unstable under fluctuating pH and environmental conditions, leading to enzyme detachment [19]. Enzyme reconstruction offers a solution to this instability, preventing enzyme leaching and enabling the continuous operation of multienzyme cascade systems. For instance, covalently conjugating enzymes to MOF‐Fe can create robust cascade reaction systems exhibiting superior stability and resistance to environmental changes compared to systems utilizing freely diffusing enzymes [20].

The use of nanozymes for parasitic disease treatment offers an emerging strategy to circumvent chemical resistance development [21]. *Trichinella spiralis* is a typical zoonotic parasite that infect both humans and animals [22]. This pathogen colonizes intestinal and muscular tissues, posing severe threats to global food safety, livestock production, and regional economies, particularly in resource‐limited settings [23, 24]. Adult worm (AD) reside in the host’s small intestine, where they mate and release newborn larvae (NBL) that penetrate the intestinal barrier, enter the bloodstream, and ultimately muscle larval (ML) cysts in striated muscle for long‐term survival [25]. Crucially, the intestinal phase represents the most active period of *T. spiralis*’s life cycle, inducing severe host inflammatory responses [26, 27]. Therefore, *T. spiralis* is a highly representative model for intestinal parasitic infections [26, 28, 29].

Herein, we designed MPG(Fe), an integrated GOx/POD cascade system synthesized by conjugating GOx to a POD‐like nanozyme, zirconium‐based MOF‐Fe, via a bifunctional PEG linker. Systematic pathogenicity assessment confirmed that MPG(Fe) significantly kills *T. spiralis*. This work pioneers a nanozyme‐based antihelminthic therapeutic leveraging the GOx/POD cascade reaction, potentially enabling broader applications of nanozymes against zoonotic intestinal parasites.

## 2. Materials and Methods

### 2.1. Materials and Instrumentation

Zirconyl chloride octahydrate (ZrOCl_2_·8H_2_O, 99.9%), ferrous (II) chloride tetrahydrate (FeCl_2_·4H_2_O, 99.99%), N‐hydroxy succinimide (NHS, 98%), N‐(3‐dimethylaminopropyl)‐N′‐ethylcarbodiimide hydrochloride (EDC·HCl, 98%), KBr (spectral grade), fluorescein isothiocyanate (FITC, 95%), and 5,5‐dimethyl‐1‐pyrroline N‐oxide (DMPO, 97%) were purchased from Aladdin Ltd. (Shanghai). 4,4^′^,4^″^,4^‴^‐(porphine‐5,10,15,20‐tetrayl) tetrakis (benzoic acid) (TCPP, 97%) and methylene blue (98%) were purchased from TCI Development Co., Ltd. (Shanghai). 2,2^′^‐azinobis(3‐ethylbenzothiazoline‐6‐sulfonic acid ammonium salt) (ABTS, 98%) and 3,3^′^,5,5^′^‐tetramethylbenzidine (TMB, 99%) were purchased from Sigma–Aldrich Ltd. Glucose (C_6_H_12_O_6_, 99.5%) was purchased Amresco Ltd. Horseradish POD (HRP, ≥300 U mg^−1^) and GOx (100–250 U mg^−1^) were purchased from Shanghai Yuanye Bio‐Technology Co., Ltd. High­ glucose Dulbecco’s modified Eagle’s medium (DMEM), penicillin­streptomycin (10,000 U mL^−1^), 3,5‐dinitrosalicylic acid (DNS), 2‐(N‐morpholino)‐ethane sulfonic acid (MES) and an Amino Acid Assay Kit were purchased from Beijing Solarbio Science & Technology Co., Ltd. Phosphate‐buffered saline (PBS, pH 7.4, 1×) and a Bradford Protein Concentration Assay Kit were purchased from Sparkjade Science Co., Ltd. A Cell Counting Kit‐8 (CCK‐8) was purchased from APExBIO Technology LLC. N,N‐dimethylformamide (DMF, 99.5%), acetic acid (CH_3_COOH, 99.5%), hydrochloric acid (HCl, 36%), hydrogen peroxide solution (H_2_O_2_, 30%), and other chemicals and reagents were purchased from Sinopharm Chemical Reagent Co., Ltd. Water was prepared using a Milli‐Q water purification system that was purchased from Millipore.

Transmission electron microscope (TEM) images were taken using a JEOL JEM‐2100F system operated using an accelerating voltage of 120 kV. Scanning electron microscope (SEM) images were obtained using a JEOL JSM‐7900F SEM system. X‐ray diffraction (XRD) patterns were obtained using an R‐AXIS RAPID system based on irradiation of samples with Cu Kα irradiation at a scan rate of 1° min^−1^ using a target voltage of 40 kV and a current set to 40 mA. UV–vis absorption spectra were recorded using a UV‐2700 UV–Vis spectrophotometer (Shimadzu, Japan). Analyses of Fourier‐transform infrared (FTIR) spectra were performed using a VERTEX 80V system (Brucker, Germany). Zeta potential data were analyzed using a Malvern NANO ZS90 instrument with a Ne laser (633 nm, 4 mW). Fluorescence microscopy images were obtained using an Olympus FV3000 confocal laser scanning microscope (CLSM).

### 2.2. Preparation of MOF‐Fe, PEG‐MOF‐Fe (MP[Fe]), and GOx‐MP(Fe) (MPG[Fe])

The TCPP‐Fe was prepared by suspending 160 mg of TCPP and 400 mg of FeCl_2_·4H_2_O in 20 mL of DMF, followed by refluxing of the mixture at 200°C for 1 h. The solid material was washed three times with water and dried to generate a black solid. Next, MOF‐525(Fe) (hereafter referred to as MOF‐Fe) was synthesized according to Kelty’s procedure with slight modifications [30] and TCPP‐Fe (94.9 mg) was dissolved in DMF (30 mL) in a 100‐mL iodine flask using sonication. In the following step, acetic acid (0.2 mL) was added, and the resulting solution was heated to 90°C and then maintained at this temperature for 18 h. The resulting nanocrystal precipitates were collected by centrifugation (27,000 × *g*, 10 min) and then subjected to solvent exchange with DMF (3 × 10 mL). The resulting MOF‐Fe solid was suspended in DMF for future use.

MOF was activated to remove unreacted organic ligands, metal salt ions, and solvent molecules present within the pores and surfaces of the material using a previously reported method [31]. Briefly, MOF‐Fe (30 mg) was dissolved in 20 mL of DMF using an ultrasound. Next, 0.75 mL of HCl (8 M) was added to the solution, and then the resulting solution was incubated at 120°C for 12 h to allow the reaction to proceed to completion. Crystals that formed were collected by centrifugation (27,000 × *g*, 10 min), and then solvent exchange with DMF was conducted (3 × 10 mL). The resulting MOF‐Fe material was suspended in DMF for future use.

To synthesized PEG‐MOF‐Fe (MP[Fe]), the MOF‐Fe was dissolved in 10 mL of DMF using ultrasound, then NH_2_─PEG─COOH (MW: 2000) was added to the solution at different mass ratios (PEG:MOF = 0.01:1, 0.05:1, 0.1:1, 0.25:1, 0.5:1, 1:1, 2:1, 4:1, and 8:1), and reactions were allowed to proceed to completion at 37°C for 6 h. Thereafter, precipitated material was collected by centrifugation (27,000 × *g*, 10 min) then subjected to solvent exchange with Milli‐Q water (3 × 1 mL). Next, reaction products were placed in Labconco CentriVap Aqueous Concentrator Systems for use in evaporating the residual water. The efficiency of covalent conjugation of NH_2_─PEG─COOH to MOF‐Fe in each reaction was determined using an Amino Acid Assay Kit (Beijing Solarbio Science & Technology Co., Ltd.).

GOx (10 mg) was dissolved in 2 mL of MES buffer (pH 6.5), then 100 μL of EDC (20 mM) was added to the tube, followed by gentle shaking of the tube at 37°C for 15 min. Subsequently, 100 μL of 20 mM NHS and 10 mL of MP(Fe) (1 mg mL^−1^) were added to the abovementioned solution, followed by incubation of the resulting solution for 1 h at 37°C. Finally, MPG(Fe) was obtained by centrifugation (15000 rpm, 10 min) followed by solvent exchange with Milli‐Q water (3 × 1 mL). Thereafter, the final GOx‐MP(Fe) (MPG[Fe]) preparation was placed in a Labconco CentriVap Aqueous Concentrator System to remove residual Milli‐Q water via evaporation. The efficiency of covalent conjugation of GOx to MP(Fe) was determined using a Bradford Protein Concentration Assay Kit (Sparkjade Biotechnology Co., Ltd.).

### 2.3. POD Mimetic Activities of MOF‐Fe and MPG(Fe)

Km and Vmax values associated with MOF‐Fe POD mimetic activity were calculated using a Lineweaver–Burk plot by surveying initial rates of reactions containing various H_2_O_2_ concentrations (ranging from 0.01 to 25.0 mM) in the presence of 2 mM TMB or various TMB concentrations (ranging from 0.0625 to 4.0 mM) in the presence of 25 mM H_2_O_2_. The POD mimetic activity of MPG(Fe) was measured by monitoring ABTS absorbance intensity changes in the presence of MPG(Fe) and H_2_O_2_ through the analysis of UV/vis spectra collected at 417 nm. The effect of pH on MPG(Fe) POD mimetic activity was studied within the pH range of 2.0–12.0. The effect of temperature on MPG(Fe) POD‐mimetic activity was investigated within the temperature range of 15–45°C. Meanwhile, different concentrations of MPG(Fe) (0, 20, or 50 μg mL^−1^) were added into 20 mL of PBS buffer (initial pH 7.4) containing 50 mg mL^−1^ glucose, and then pH value measurements were taken using a real‐time pH meter.

### 2.4. •OH Generation Ability of MPG(Fe)

DMPO served as a trapping probe for use in electron paramagnetic resonance (EPR) spectroscopic monitoring of •OH generation ([H_2_O_2_] = 100 μM, [MPG(Fe)] = 100 μg mL^−1^). In addition, •OH generation was monitored via fluorescence spectroscopy, a method based on terephthalic acid (TA) capture of •OH that generates a 2‐hydroxy TA product that uniquely fluoresces when excited with light of wavelength approximately 435 nm. Briefly, HAc–NaAc buffer solutions (pH 6.0, 10 mM) containing TA (0.5 mM), H_2_O_2_ (1 mM), and/or MOF‐Fe (100 μg mL^−1^) were prepared, and then reaction tubes containing TA, TA with H_2_O_2_, TA with MOF‐Fe, and TA with MOF‐Fe and H_2_O_2_ were prepared. Reactions were allowed to proceed for 12 h, and then reaction tubes were centrifuged. Thereafter, the fluorescence spectra of the resulting supernatants were obtained and analyzed.

### 2.5. Glucose Consumption

MPG(Fe) solutions at different concentrations (50, 100, and 200 μg mL^−1^) were added to a 1 mg mL^−1^ glucose solution, and then the DNS method was used to monitor the glucose concentration during the reaction. In brief, at different time points, 0.2 mL aliquots of the abovementioned solutions were removed and centrifuged. Next, 0.8 mL of DNS was added to each sample, and then tubes containing samples were heated at 100°C for 5 min, quickly cooled in a water bath and left there for 20 min. Absorbance intensity changes were monitored via UV/vis spectroscopy conducted at 540 nm.

### 2.6. H_2_O_2_ Generation

H_2_O_2_ generation was assessed by conducting ABTS UV–vis absorbance measurements at 734 nm in the presence of HRP (HRP). In brief, first glucose (50 mg mL^−1^) and MPG(Fe) (50, 100, and 200 μg mL^−1^) were mixed. Next, 200 μL aliquots of the solution were removed at different time points, then 50 μL of HRP (1 U mL^−1^), 850 μL of HAc‐NaAc buffer (pH 6.0, 10 mM), and 100 μL of ABTS (2 mM) were added to each aliquot. After reactions proceeded for 10 min, ABTS absorbance intensity changes of reactions were monitored by recording UV/vis spectra at 734 nm in order to quantify the sample H_2_O_2_ concentrations based on a standard curve generated using the same method.

### 2.7. *In Vitro* Cytotoxicity of MOF‐Fe and MPG(Fe)

IPEC‐J2 cells were used for in vitro experiments. Cells were maintained in high­glucose DMEM containing 10% fetal bovine serum and 1% penicillin­streptomycin (10,000 U mL^−1^) in an atmosphere of 5% CO_2_ at 37°C.

IPEC‐J2 cells (5 × 10^3^ cells well^−1^) were cultured in 96­well plates (Jet Bio‐Filtration Co., Ltd.) for 24 h at 37°C. Next, MOF‐Fe or MPG(Fe) was added to wells of plates to achieve final concentrations of 1.5, 3, 6, 12, 25, 50, or 100 μg mL^−1^. Thereafter, plates were incubated for an additional 6, 12, or 24 h, and then cell viability was assessed using the CCK­8 method.

### 2.8. Ethical Treatment of Animals and *T. spiralis* Organisms

In this study, 6‐ to 8‐week‐old male Wistar rats weighing approximately 200 g and 6‐ to 8‐week‐old male BALB/c mice weighing approximately 20 g were used in experiments. Animals were fed normal chow and distilled water and housed in an animal room where the temperature was maintained at 24 ± 2°C, humidity at 55% ± 5% and lighting set to a 12 h day/12 h night cycle. All animal experiments were performed according to regulations of the Administration of Affairs Concerning Experimental Animals in China using protocols that were approved by the Institutional Animal Care and Use Committee of Jilin University (No. SY202511035). *T. spiralis* (ISS534) parasites were provided by the Institute of Zoonosis, Jilin University.

### 2.9. Preparation of *T. spiralis* Adult (AD) Worms

ADs were collected according to the method previously studied from small intestines of 8‐ to 10‐week‐old Wistar rats that had been inoculated 3 days earlier with 5000 larvae [32]. Briefly, intestines were washed, then intestinal samples were suspended in saline containing 2× penicillin–streptomycin, and then, samples were incubated at 37°C for 2 h. Thereafter, the small intestine tissue fragments and the upper layer of liquid were carefully removed, and then the remaining liquid containing ADs was transferred to a flat dish. Next, ADs were rinsed repeatedly with saline to remove small intestine debris. Next, washed ADs were resuspended and cultured in RMPI 1640 medium containing 2× penicillin–streptomycin until needed for experiments.

### 2.10. Preparation of NBL

After 8‐ to 10‐week‐old Wistar rats were inoculated with 5000 larvae, ADs were collected from small intestines of rats 5 days later using the abovementioned procedure. Thereafter, collected ADs were cultured in RMPI 1640 medium containing 2× penicillin–streptomycin and 10% FBS at 37°C in 5% CO_2_ for 24 h. NBLs were separated from ADs via filtration using a Falcon Cell Strainer (100 μm hole size) and incubated in RMPI 1640 medium (containing 2× penicillin–streptomycin) until subjected to further analysis.

### 2.11. Effects of MPG(Fe) Treatment on ADs and NBLs *In Vitro*

ADs (10 to 20 worms well^−1^) were cultured in 12 ­well plates (Jet Bio‐Filtration Co., Ltd.) and NBLs (30 to 40 larvae per well) were cultured in 96­ well plates (Jet Bio‐Filtration Co., Ltd.). ADs were cultured in PBS or MPG(Fe) at different concentrations (5, 10, 25, 50, or 100 μg mL^−1^) in wells of 12­ well plates in a total volume/well of 2 mL of PBS (pH 7.4) containing 5% glucose. For NBLs, larvae in wells of 96‐well plates were treated with PBS or MPG(Fe) at different concentrations (5, 10, 50, or 100 μg mL^−1^) in a total volume/well of 0.2 mL of PBS (pH 7.4) containing 5% glucose. Numbers of viable and dead ADs and NBLs in tissues were determined by viewing Hoechst‐ and propidium iodide (PI)‐stained tissues under a CLSM. In addition, numbers of ADs were determined to assess the effects of free GOx and MOF‐Fe exposures on AD viability.

### 2.12. Effects of MPG(Fe) Treatment on ADs *In Vivo*

BALB/c mice (aged 6–8 weeks) were inoculated with 500 larvae and injected intraperitoneally with low‐dose (2.5 mg kg^−1^), medium‐dose (7.5 mg kg^−1^), high‐dose (15 mg kg^−1^) MPG(Fe), or PBS at 6, 12, and 24 h after larval inoculation. At 30‐h after inoculation, ADs were collected using the abovementioned method, and then dead larvae were counted.

### 2.13. Evaluation of MPG(Fe) Biosafety and Toxicity *In Vivo*

To evaluate MPG(Fe) therapeutic safety and toxicity, 6‐ to 8‐week‐old BALB/c mice were randomly assigned to two groups for short‐term (6, 12, 24, and 30 h) and long‐term (1, 3, 5, and 7 days) MPG(Fe) administration. Subgroups of mice within the two groups were treated with PBS or low‐dose MPG(Fe) (2.5 mg kg^−1^), medium‐dose MPG(Fe) (7.5 mg kg^−1^) or high‐dose MPG(Fe) (15 mg kg^−1^). All animal studies were performed according to protocols that were approved by the Institutional Animal Care and Use Committee of Jilin University.

### 2.14. Blood Biochemical Index Analysis

Blood samples were collected from eye orbital veins of mice in both groups using 1 mL blood collection microtubes containing ethylenediaminetetraacetic acid (EDTA) anticoagulant. White blood cells (WBCs), lymph (lymphocyte), MONs, neutrophilic granulocyte (Gran), neutrophilic Gran%, red blood cells (RBCs), hemoglobin (HGB), and blood platelet (PLT) values were obtained for each blood sample using a Mindray BC‐2800vet Veterinary Automated Hematology Analyzer.

### 2.15. Flow Cytometric Analysis of Mouse Splenic T and B Cells

Spleens were excised from mice of the two groups, then spleens were pulverized and filtered (100‐mesh) and spleen cells were resuspended in PBS. RBCs were removed after spleen cells were centrifuged at 260 × *g* for 10 min by discarding the pellet and then resuspending nonpelleted cells in Red Blood Cell Lysis Buffer (Solarbio) to lyse residual RBCs. The remaining intact cells were washed 2–3 times with PBS and then adjusted to a cell count per volume of 5 × 10^6^ cells mL^−1^. Suspended T and B cells were prepared for flow cytometric analysis by incubating them with a phycoerythrin (PE)‐conjugated antibody that specifically binds to the CD45 leukocyte marker present on all hematopoietic cells except PLTs and erythrocytes (BioLegend 103105), an allophycocyanin (APC)‐conjugated antibody that specifically binds to the CD45R marker found on surfaces of B cells and subsets of T cells and NK cells (BioLegend 103211) or a FITC‐conjugated antibody that specifically binds to the T cell receptor component CD3e present on T cell surfaces (BD Pharmingen 553061). After cells were incubated with the abovementioned antibodies, they were washed with PBS, and then 10^6^ cells from each sample were analyzed using a BDverse flow cytometer. All data sets were analyzed using FlowJo 10 data analysis software (FLOWJO, LLC).

### 2.16. Immunofluorescence Staining

Spleens collected from mice of the two groups that received different treatment doses were collected and fixed in a 4% paraformaldehyde solution. Briefly, fixed spleens were hydrated by immersion in xylene, followed by stepwise immersion in solutions of gradient alcohol. Thereafter, rehydrated spleens were boiled in an EDTA solution (pH = 8.0, Solarbio, Beijing, China) for 15 min to ensure that tissue antigens would be accessible to immunofluorescence staining reagents. After hydrated tissue sections were cooled to room temperature, 0.05% trypsin was added to sections, and then trypsinization of sections was allowed to proceed for 10 min to destroy auto‐fluorescent fibrous tissue. Next, the spleens were embedded in paraffin and then sectioned for immunofluorescence staining. Direct immunostaining was conducted to label T cells and B cells with FITC‐conjugated anti‐mouse CD3e antibody (BD Pharmingen 553061) and B cells with PE anti‐mouse CD45 antibody (BioLegend 103105). Nuclei were stained with Hoechst 33342 (Solarbio) to facilitate cellular localization. Images were obtained using confocal laser microscopy.

### 2.17. Hematoxylin and Eosin (H&E or HE) Staining

Mice belonging to groups receiving different treatment doses or no treatment were sacrificed at predetermined times. Thereafter, hearts, livers, spleens, lungs, and kidneys of mice were collected. The collected organs were fixed in 4% paraformaldehyde solution for 24 h, and then the organs were embedded in paraffin after dehydration. The middle portion of each embedded organ was cut transversely into 4 μm‐thick sections, and then sections were processed for routine H&E staining, which was conducted using a kit (HE Staining Kit, Solarbio, Beijing, China). Slides were examined via light microscopy (Olympus, Tokyo, Japan).

### 2.18. Statistical Analysis

All data in this work are expressed as the mean ± SD. Comparisons between groups were conducted using *t*‐tests. One‐way analysis of variance (ANOVA) was conducted to assess the statistical significance of differences in results obtained between groups receiving different treatments, with variations deemed significant at  ^∗∗∗∗^ *p* < 0.0001,  ^∗∗∗^ *p* < 0.001,  ^∗∗^ *p* < 0.01, and  ^∗^ *p* < 0.05. All statistical calculations and computational data analysis were performed using GraphPad Prism 8.0 software (GraphPad Software Inc., CA, USA).

## 3. Results

### 3.1. Synthesis and Characterization of MPG(Fe)

MPG(Fe) synthesis is a three‐step process (Figure 1a). First, the TCPP‐Fe and MOF‐Fe (MOF‐525[Fe]) ligand was synthesized as previously described and exposed to NH_2_─PEG─COOH dissolved in DMF at room temperature for 6 h. Using a “solvent‐assisted ligand incorporation” functionalization strategy, unoccupied MOF‐Fe terminal hydroxy groups were linked to the ─COOH group of NH_2_─PEG─COOH to generate PEG─MOF─Fe (MP[Fe]) then GOx was covalently linked to PEG‐MOF‐Fe via a reaction involving EDC and NHS [30, 33]. MPG(Fe) (MPG without Fe) was fabricated using the same method, except that TCPP served as the MOF ligand. SEM and TEM images revealed an ellipsoid MPG(Fe) shape with dimensions of approximately 30 nm (Figure 1b,c). Comparisons of MOF‐Fe and MPG(Fe) particle sizes revealed that the MPG(Fe) particle size was greater overall, thus indicating that successful surface modifications involving additions of PEG and GOx to MOF‐Fe had been achieved (Figure S1). Notably, PEG modification increased MOF particle dispersity and prevented severe MOF‐Fe particle aggregation from occurring in electrolyte solutions [34, 35]. UV–vis absorption spectra revealed blue shifts in the Soret band region (from 410 to 405 nm) and the Q band region, with shifts in the latter region accompanied by a decrease in peak number from three peaks (528, 556, and 655 nm) to two peaks (582 and 627 nm), thus demonstrating that TCPP metallization with iron had occurred (Figure S2) [36]. These spectral peak changes were consistent with Yaghi’s results showing that peak changes appeared in Q and Soret band regions after MOF metallization with Fe had occurred.

Figure 1Structure characterization of MPG(Fe). (a) Schematic diagram of synthetic steps for MPG(Fe). (b) SEM image of MPG(Fe). (c) TEM image of MPG(Fe). (d) UV–Vis spectra. (e) XPS spectra of Zr 3d orbit for MPG(Fe) and MOF‐Fe. (f) EDS element mapping of MPG(Fe), Fe (blue) and S (purple and pink).

**Figure:**
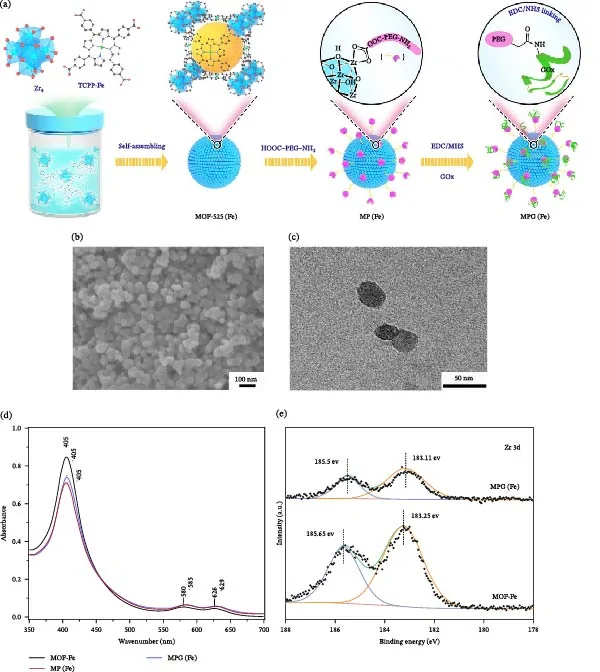

**Figure:**
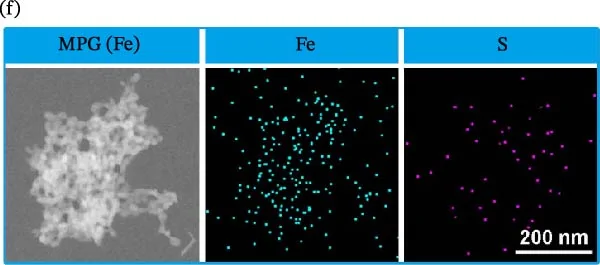

To further explore the effects of PEG and GOx modifications on MOF‐Fe porphyrin ligands, 2M NaOH was used to digest MOF‐Fe, MP(Fe), and MPG(Fe). The results of these experiments revealed that after MOF‐Fe was modified with PEG (MP[Fe]) and PEG and GOx (MPG[Fe]), no significant spectral changes in porphyrin absorption peaks were detected within Soret and Q band regions (Figure 1d). Furthermore, XRD pattern analysis confirmed Yaghi’s reported results, showing that MOF‐Fe particles with and without PEG and GOx modifications were isostructural, thus confirming that these modifications did not alter the MOF‐Fe structure. Nevertheless, intensities of diffraction peaks of MP(Fe) and MPG(Fe) were weaker than those of corresponding peaks obtained for unmodified MOF‐Fe (Figure S3), thus showing that surface modifications of MOF‐Fe led to altered diffraction signal intensities [36]. Meanwhile, the coordination state of Zr within MPG(Fe) and the existence of a binding interaction between NH_2_─PEG─COOH and the MOF‐Fe Zr_6_ cluster were both confirmed using X‐ray electron spectroscopy (XPS) (Figure 1e). Specifically, two characteristic MPG(Fe) peaks observed at 183.11 and 185.5 eV corresponding to Zr 3d_5/2_ and 3d_3/2_ energy levels, respectively, were shifted to lower values by 0.13 eV and 0.15 eV, respectively, relative to the corresponding MOF‐Fe eV values. These results indicate that Zr atoms within Zr_6_ cluster nodes carry more charge than Zr atoms outside of such nodes and also indicate that the Zr charge within these nodes was redistributed; thus, the ─COOH group of NH_2_─PEG─COOH was successfully linked to the unsaturated site of the MOF‐Fe Zr_6_ cluster. Energy‐dispersive spectrometry (EDS) elemental mapping of MPG(Fe) further confirmed the presence of Fe and S within the complex as evidence that metallization of the MOF and GOx had occurred (Figure 1f). Taken together, all of the abovementioned results indicated that we successfully synthesized MPG(Fe) and that it possessed a stable structure. By contrast, MOF‐Fe was observed to disperse poorly in aqueous solutions, resulting in rapid aggregation, while zeta potential analysis results indicated improved dispersion of MOF‐Fe in an aqueous solution after PEG and GOx molecules were attached to its surfaces. Importantly, improved dispersion would enhance MOF‐Fe bioavailability and thus increase its usefulness for biological applications (Figure S4).

To determine the optimal PEG/MOF‐Fe ratio for MPG(Fe) synthesis, the concentration of PEG covalently conjugated to MOF‐Fe was analyzed at different input PEG:MOF‐Fe ratios by quantifying the ─NH_2_ content. The results revealed that the amount of covalently conjugated PEG increased with the increasing input ratio and tended to plateau once the PEG/MOF‐Fe ratio exceeded 1:1. This plateau corresponded to approximately 0.186 mg of NH_2_─PEG─COOH (MW: 2000) covalently conjugated per 1 mg of MOF‐Fe, which was therefore defined as the optimal PEG/MOF‐Fe ratio for MPG(Fe) synthesis (Figure S5). In order to optimize PEG‐induced direct adsorption of GOx to MOF‐Fe, GOx adsorption to MOF‐Fe was assessed using reaction solutions with different pH values (Figure S6). The results of these experiments revealed that at pH 6.0 in the absence of PEG, the quantity of GOx that adsorbed to MOF‐Fe was only about 30% of that which adsorbed to MOF‐Fe at pH 7.0, while no GOx adsorption was observed when the pH fell below 5.0. In contrast, adsorption of GOx to MOF‐Fe in the presence of PEG was not affected by pH changes, thus indicating that PEG stabilized the binding interaction between GOx and MOF‐Fe within MPG(Fe) to facilitate dual‐enzyme cascade catalysis. The functionalized PEG (NH_2_─PEG─COOH) served as a bridge to support the formation of a cascade complex based on an acid–base chemical interaction between the hydroxyl group of the MOF‐Fe node and the carboxyl group of the natural enzyme GOx [33]. Importantly, PEG‐induced immobilization of GOx increased the stability of the cascade complex to promote greater •OH production and glucose consumption that would markedly reduce *T. spiralis* survival. Furthermore, addition of PEG to the mixture of MOF‐Fe and GOx increased the solubilities of the resulting cascade complex MPG(Fe) and of MOF‐Fe.

### 3.2. MPG(Fe) Cascade Reaction

**Figure 2:**
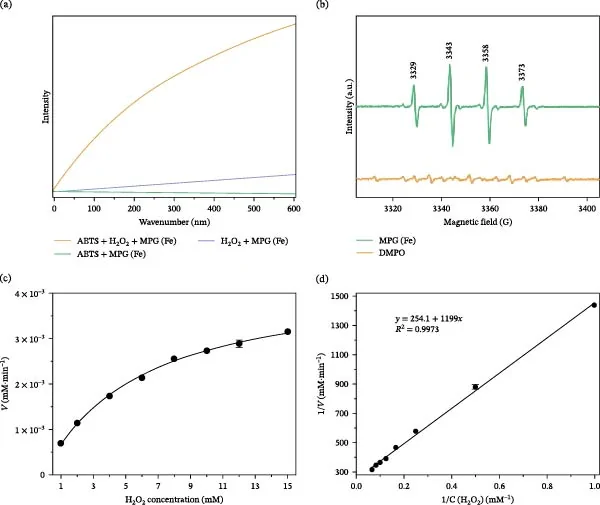
The peroxidase‐like activity of MPG(Fe). (a) UV–Vis absorbance spectra of ABTS solutions with the addition of (1) 10 μg mL^−1^ MPG(Fe), 10 mM H_2_O_2_, 1 mM ABTS, pH = 4.0; (2) 10 μg mL^−1^ MPG(Fe), 10 mM H_2_O_2_; and (3) 10 μg mL^−1^ MPG(Fe), 1 mM ABTS. (b) ESR spectra obtained with the addition of 10 μg mL^−1^ MPG(Fe) and 10 mM H_2_O_2_. (c) The Michaelis–Menten fitting curve of initial ^·^OH generation velocities against H_2_O_2_ concentrations. (d) The Lineweaver–Burk fitting (double reciprocal) of the Michaelis–Menten fitting curve.

After we achieved the successful synthesis of MPG(Fe), we evaluated its POD‐like activity by measuring changes in ABTS absorbance at 734 nm. The results of this analysis showed that both MPG(Fe) and H_2_O_2_ were required for catalytic oxidation of ABTS to occur, that reactive oxygen species (ROS) were generated during this process, and that MPG(Fe) possessed POD‐like catalytic ability (Figure 2a). To determine whether •OH radicals were generated by the MPG(Fe)‐catalyzed POD reaction, DMPO was used to trap the radicals, and then the DMPO/•OH ratio was determined using electron spin resonance spectroscopy (ESR). ESR results revealed the presence of a typical DMPO/•OH 4‐peak profile with relative peak intensities of 1:2:2:1 in ESR spectra obtained in the presence of H_2_O_2_ (Figure 2b). In addition, results obtained from experiments based on TA trapping of •OH to generate the strongly fluorescent product 2‐hydroxy TA revealed a strong fluorescence signal in the spectra obtained in the presence of H_2_O_2_ (Figure S7). In summary, the MPG(Fe) POD was shown to catalyze the production of •OH and exhibited maximal activity at a pH of 4.0 and no activity when the pH exceeded 10 (Figure S8). Moreover, MPG(Fe) catalytic capacity increased with increasing temperature (Figure S8). As compared to active centers of native PODs, the MPG(Fe) active center is less affected by temperature changes, since it contains a MOF formed from iron porphyrins that are coordinated by the Zr_6_ cluster that together stabilize the MOF structure. Nevertheless, with increasing temperature, MOF molecular movements become more vigorous, thus increasing the chance that the substrate will collide with an active center porphyrin iron per unit time to increase the catalytic rate while weakening MOF structural integrity. Analysis of ABTS steady‐state reaction kinetics as a function of H_2_O_2_ concentration revealed typical Michaelis–Menten kinetics (Figure 2c). After the double reciprocal plot was fitted to the Michaelis–Menten equation, the Michaelis constant (*K*_M_) and maximum reaction rate (*V*_max_) were calculated and found to be 5.81 mM and 7.223 × 10^−8^ M S^−1^, respectively (Figure 2d). Similarly, analysis of reaction kinetics based on ABTS as a substrate produced values of *K*_M_ and *V*_max_ of 0.031 mM and 7.205 × 10^−8^ M S^−1^, respectively (Figure S9). Taken together, the abovementioned results confirm that MPG(Fe) synthesized in this study possessed greater affinity for H_2_O_2_ as compared to previously reported nanozymes, which, in turn, facilitated MPG(Fe) binding to the H_2_O_2_ substrate and accelerated catalytic generation of •OH from H_2_O_2_ (Table S1).

**Figure 3:**
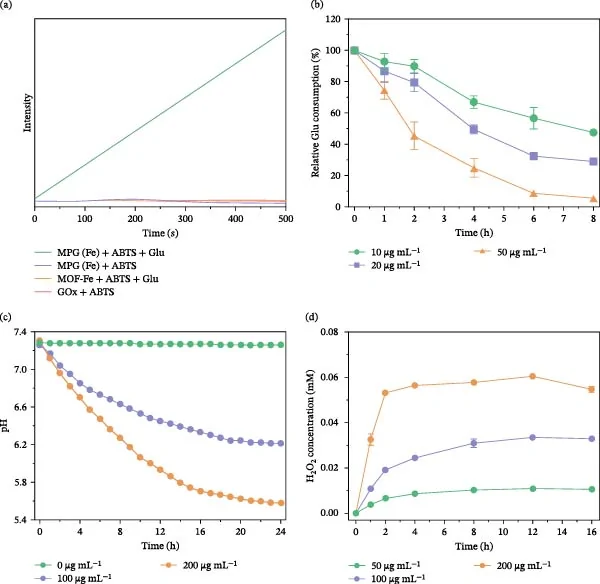
The MPG(Fe) cascade activity effect by glucose, pH, and H_2_O_2_ concentration. (a) Time‐dependent absorbance changes of ABTS at 734 nm (green line: with 10 μg mL^−1^ MPG(Fe), 1 mM ABTS, and 1 mg mL^−1^ glucose; purple line: with 10 μg mL^−1^ MPG(Fe) and 1 mM ABTS; orange line: with 10 μg mL^−1^ MOF‐Fe, 1 mM ABTS and 1 mg mL^−1^ glucose; and pink line: 1 mM ABTS, 1 μg mL^−1^ GOx). (b) Percentage of glucose consumption in the presence of 10 μg mL^−1^ MPG(Fe) (green line), 20 μg mL^−1^ MPG(Fe) (purple line), and 50 μg mL^−1^ MPG(Fe) (orange line). The initial concentration of glucose was 50 mg mL^−1^. The change of (c) pH value and (d) H_2_O_2_ concentration with the addition of 0, 100, and 200 μg mL^−1^ MPG(Fe) in the glucose solution 50 mg mL^−1^ and 1 mg mL^−1^, respectively.

To verify the cascade catalytic capability of MPG(Fe), glucose was used as a reactant to initiate the cascade catalytic process, and then time‐dependent kinetics of the reaction were analyzed, with results shown in Figure 3a. It is clear that the presence of both MPG(Fe) and glucose was required for catalytic oxidation of ABTS to occur, since no catalysis was observed in the presence of MOF‐Fe without GOx loading, as expected. We also used DMPO to capture the free radicals generated during the reaction, resulting in a significant hydroxyl radical signal (Figure S10). Importantly, glucose consumption led to downstream production of glucuronic acid and H_2_O_2_, which are often viewed as key indicators of GOx activity. Moreover, an examination of glucose consumption using the DNS method revealed that the glucose concentration decreased rapidly when MPG(Fe) was present and that a higher concentration of MPG(Fe) evoked a more rapid glucose consumption. Thus, the glucose consumption rate was highly dependent on the MPG(Fe) concentration (Figure 3b and Figure S11). Meanwhile, pH changes that occur as glucuronic acid is produced by the reaction between glucose and MPG(Fe) revealed that the pH value continually dropped over 18 h, thus confirming that gluconic acid was generated via a continuous reaction process (Figure 3c). Furthermore, changes in the H_2_O_2_ concentration that were detected using the ABTS method revealed that use of MPG(Fe) at a concentration of 200 μg mL^−1^ caused the H_2_O_2_ concentration in the solution to reach 55 × 10^−6^ M within 2 h, thus indicating that rapid oxidation of glucose occurred (Figure 3d and Figure S11). Therefore, the abovementioned findings demonstrated that MPG(Fe) can exert a POD/GOx cascade catalytic effect.

### 3.3. In Vitro MPG Effects on the IPEC‐J2 Cell Line and T. spiralis

**Figure 4:**
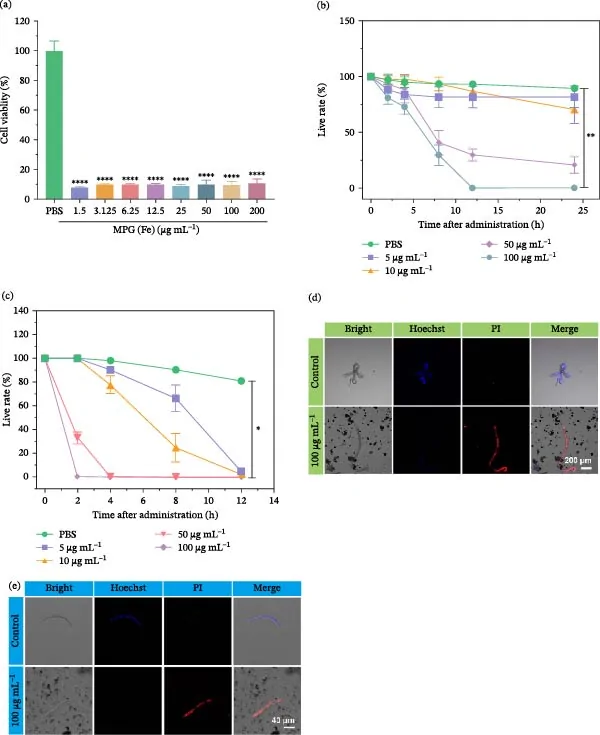
(a) IPEC‐J2 cell viabilities of MPG(Fe) with different concentration (0–200 μg mL^−1^). Live rate of MPG(Fe) with a different concentration on AD (b) and NBL (c) in vitro. The fluorescence images of AD (d) and NBL (e) were incubated with PBS and 100 μg mL^−1^ MPG(Fe) after Hoechst‐PI staining for live/death detection. ( ^∗^ *p* < 0.05,  ^∗∗^ *p* < 0.01, and  ^∗∗∗∗^ *p* < 0.0001).

MOF‐Fe cytotoxicity for cells of the porcine enterocyte‐derived cell line IPEC‐J2 was assessed using the CCK‐8 assay, with no significant toxicity observed in vitro (Figure S12). After loading with GOx, the inhibitory effect of MPG(Fe) on IPEC‐J2 cell viability was significantly higher than that of MOF‐Fe, and the cell viability decreased to 10% of the PBS group (Figure 4a). To investigate the underlying mechanism for the observed MPG(Fe)‐induced reductions in cell viability, equimolar doses of MPG(Fe) and GOx (Figure S12) were compared for their effects on cell viability. As compared with the PBS group, molar equivalent doses of MPG(Fe) and GOx significantly reduced cell viability, with the trend in the GOx effect following the same trend observed for the MPG(Fe) effect. Therefore, the main explanation for why MPG(Fe) reduced IPEC‐J2 cell viability was that glucose, an energy source provided by the culture medium, was oxidized by its component GOx and thus was no longer available in sufficient amounts to support normal IPEC‐12 growth and reproduction. In addition, in vitro experiments confirmed that MPG(Fe) oxidized glucose within the intestinal cell environment to thereby reduce glucose levels, while also inhibiting glucose uptake by *T. spiralis* to impede parasite growth and development.

According to the *T. spiralis* lifecycle, a reduction in the number of adult worms (AD) can effectively reduce the yield of NBL to thereby reduce the adverse effects of trichinellosis on host health. Notably, the AD survival rate decreased with increasing MPG(Fe) concentration and thus was highly dependent on the MPG(Fe) concentration. Indeed, ADs exposed in vitro to MPG(Fe) at a concentration of 100 μg mL^−1^ were completely killed after 12 h (Figure 4b). In addition, in vitro experiments conducted to compare the anti‐AD activities of MPG(Fe) and MOF‐Fe, H_2_O_2_, and GOx revealed no anti‐AD effect when ADs were exposed to MOF‐Fe in the absence of GOx covalent loading (Figure S13). Interestingly, although low concentrations of H_2_O_2_ did not inhibit AD viability, all ADs died when exposed to a high H_2_O_2_ concentration above 1 mM, thus demonstrating that ADs could not tolerate exposure to a high H_2_O_2_ concentration (Figure S13). Meanwhile, treatment of ADs with GOx at a molar dose that was equivalent to a lethal MPG(Fe) dose did not affect AD survival (Figure S13). Thus, this result indicated that AD tolerance to H_2_O_2_ depends on H_2_O_2_ concentration, such that the concentration of H_2_O_2_ attained during GOx catalysis in the absence of MPG(Fe) was not sufficient to reduce AD survival, since ADs possess their own antioxidant system that can degrade H_2_O_2_ when present at low concentrations [37]. By contrast, the GOx‐loaded MPG(Fe) synthesized in this study directly converted glucose into more toxic hydroxyl radicals that exerted a significantly greater anti‐AD effect than that exerted by H_2_O_2_, as evidence that hydroxyl radicals generated by the degradation of H_2_O_2_ most markedly reduced AD survival. Similar MPG(Fe) effects on NBL survival were observed, whereby the NBL survival rate decreased with increasing MPG(Fe) concentration. Furthermore, a mortality rate above 95% was observed after 12‐h exposure of NBL to MPG(Fe) at a concentration of 5 μg mL^−1^, while 100% mortality was observed after 2‐h exposure of NBL to MPG(Fe) at a concentration of 100 μg mL^−1^ (Figure 4c). We also analyzed the anti‐NBL effects of MOF‐Fe, H_2_O_2_, and GOx in vitro. As consistent with results obtained for ADs, no anti‐NBL effect was observed in the presence of MOF‐Fe without covalently loaded GOx (Figure S14). However, low concentrations of H_2_O_2_ did not reduce NBL viability unless the concentration of H_2_O_2_ exceeded 0.1 mM due to the immaturity of the NBL antioxidant system that endowed NBLs with lower tolerance to H_2_O_2_ than that of ADs (Figure S14). Moreover, a GOx molar equivalent dose to that of a lethal MPG(Fe) dose did not affect NBL survival, as consistent with results obtained for ADs (Figure S14). To confirm these results, we used CLSM to investigate AD and NBL survival status after exposure to different concentrations of MPG(Fe). PI staining confirmed that complete killing was achieved at 100 µg mL^−1^ MPG(Fe) for ADs and within 2 h for NBLs. Also, damage of *T. spiralis* nuclei caused by exposure of the organisms to hydroxyl radicals produced in the presence of MPG(Fe) prevented Hoechst staining of AD and NBL nuclei (Figure 4d,e and Figures S15 and S16).

In summary, MPG(Fe) has significant anti‐AD and NBL effects in vitro, while avoiding the oxidative damage caused by excessive H_2_O_2_ concentration and reducing the damage caused by *Trichinella spiralis* infection. Additionally, MPG(Fe) treatment reduced *T. spiralis* glucose uptake from the environment as compared to the corresponding effects of other treatments, thus indicating that MPG(Fe) may consume glucose required by enteric *T. spiralis* organisms to block parasite growth and development within the host [38]. Furthermore, hydroxyl radicals (•OH) produced by MPG(Fe) negatively impacted intestinal *T. spiralis* reproductive success and survival.

### 3.4. Effects of MPG(Fe) *In Vivo*

**Figure 5:**
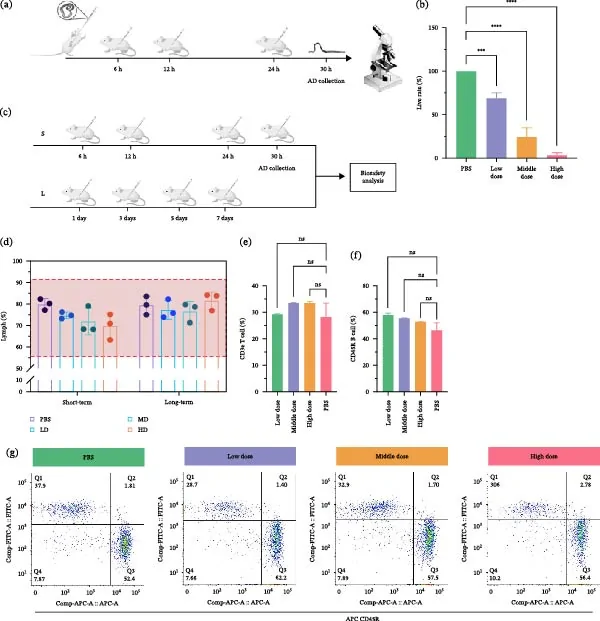
(a) Timeline of anti‐AD by MPG(Fe) with PBS, low dose (2.5 mg kg^−1^), middle dose (7.5 mg kg^−1^), and high dose (15 mg kg^−1^) in vivo. (b) Live rate with PBS and each treatment group on AD in vivo (*N* = 3). (c) Timeline of the biosafety experiment procedures. (d) Serum biochemistry (lymph%) results were obtained from mice injected with each treatment group and PBS. Flow cytometry analysis for immune cells (g) and statistical analysis of T cells (e) and B cells (f) for the short‐term administration of MPG(Fe) with different concentrations ( ^∗∗∗^ *p* < 0.001;  ^∗∗∗∗^ *p* < 0.0001; ns, nonsignificant).

The in vitro results described above prompted us to investigate MPG effects in vivo in order to better understand the effects of MPG(Fe) on ADs. At 6, 12, and 24 h after inoculation of mice with *T. spiralis* ML, low‐dose (2.5 mg kg^−1^), medium‐dose (7.5 mg kg^−1^), and high‐dose (15 mg kg^−1^) MPG(Fe) were injected into mice. At 30 h after *T. spiralis* inoculation, ADs were collected from small intestines of mice, then counted and used to calculate AD mortality (Figure 5a). The results of these experiments revealed that AD numbers gradually decreased as MPG(Fe) concentration was increased and thus were inversely correlated with the MPG(Fe) dose (Figure S17). Notably, the AD survival rate that received a high dose of MPG(Fe) (15 mg kg^−1^) was only 3.53% (Figure 5b), which indicates that in addition to its in vitro anti‐AD effect, MPG(Fe) exerted an obvious anti‐AD effect in vivo.

**Figure 6:**
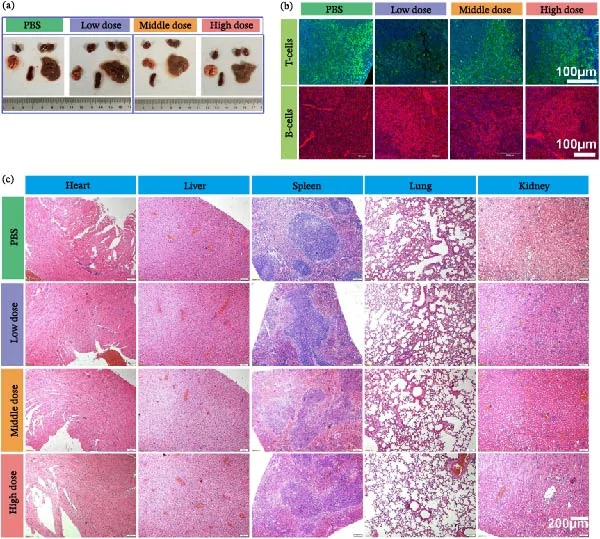
Biocompatibility evaluation of MPG(Fe). (a) Photograph of organs collected from different groups of mice after short‐term administration of MPG(Fe). (b) Immunofluorescence analysis of splenic tissue T cells and B cells with PBS and middle dose (7.5 mg kg^−1^) of MPG(Fe) by short‐term administration. (c) Representative images of H&E staining of heart, liver, spleen, lung, and kidney with PBS and middle dose (7.5 mg kg^−1^) of MPG(Fe) by short‐term administration (*N* = 3).

Biosafety and toxicity are essential characteristics used to evaluate the potential usefulness of new nanomaterials for clinical applications. Considering the lifespan of *T. spiralis*, short‐term injections (6, 12, 24, and 30 h) and long‐term injections (1, 3, 5, and 7 days) were administered to mice in order to evaluate the biological safety of low‐dose (2.5 mg kg^−1^), medium‐dose (7.5 mg kg^−1^), and high‐dose (15 mg kg^−1^) MPG(Fe), with the biosafety timeline outlined in Figure 5c. After mice received short‐term and long‐term courses of MOF‐Fe injections, whole blood was drawn from eye socket veins and subjected to biochemical analysis to detect changes in blood cell populations and levels of biochemical indicators. The results showed that after MPG(Fe) injections, no significant differences were observed in levels of lymphocytes (lymph) (Figure 5d), thus indicating that MPG(Fe) administration did not induce an immune inflammatory response in mice. Also, no effects of short‐term or long‐term MPG(Fe) injections on HGB level and counts of RBCs, PLTs, leukocytes (WBC), MONs, Grans, and mean corpuscular volume (MCV) injection were observed, thus indicating that normal physiological and biochemical functions were not affected by MPG(Fe) treatment (Figure S18). To further investigate immunological effects of MPG(Fe), flow cytometric analysis was conducted to determine whether short‐term and long‐term treatments with low‐dose (2.5 mg kg^−1^), medium‐dose (7.5 mg kg^−1^), and high‐dose (15 mg kg^−1^) MPG(Fe) affected T cell and B cell levels (Figure 5e–g and Figure S19). The results indicated a lack of statistically significant differences in T cell and B‐cell levels between the control group and short‐term and long‐term MPG(Fe) treatment groups as evidence of MPF(Fe) safety. Also, the size and shape of major organs (heart, liver, spleen, lung, and kidney) does not show typical changes compared with the PBS group and different does groups (Figure 6a). In addition, results of immunofluorescence analysis confirmed that MPG(Fe) exerted no obvious effect on T and B cell levels in spleen tissues of mice receiving short‐term and long‐term MPG(Fe) treatments (Figure 6b and Figure S20). Furthermore, H&E staining of hearts, livers, spleens, lungs and kidneys of mice revealed no pathological changes after short‐term and long‐term administration of different MPG(Fe) doses, thus demonstrating that MPG(Fe) is safe and nontoxic when administered to healthy mice (Figure 6c and Figure S21). These results also align with results showing that no obvious injury or inflammation was triggered by MPG(Fe) treatment, as evidenced by the normal appearance of toxin‐susceptible organs (heart, liver, spleen, lung, and kidney). Taken together, these results indicate that MPG(Fe) shows no detectable acute toxicity in uninfected mice over 7 days, and we emphasize that long‐term safety and safety in infected hosts remain to be determined in future studies.

## 4. Discussion

Nanozymes leverage exceptional physicochemical properties, low cost, and high stability to overcome limitations inherent in natural enzymes [39, 40]. These synthetic enzymes have demonstrated therapeutic efficacy against diverse pathologies, including tumors, bacterial infections and inflammatory disorders [41–44]. Here, we report MPG(Fe), a hybrid cascade system integrating natural GOx with a POD‐mimetic nanozyme. This platform represents a novel strategy for treating trichinellosis and other zoonotic intestinal parasitic diseases.

During the *T. spiralis* life cycle, it dynamically shifts energy sources across developmental stages and infection sites (intestinal and muscle tissue). Upon entering the intestinal environment, ML rapidly initiate growth and development, fueled primarily by endogenous glycogen reserves synthesized in prior parasitized tissues [45, 46]. Critically, intestinal adults face rapid mortality once these glycogen stores deplete unless they absorb substantial exogenous glucose to sustain energy demands. This glucose dependence is evidenced by in vitro studies, adult worms perish within 12 h without exogenous glucose [38], confirming glucose as essential for parasitism and development.

MOF‐Fe comprises octahedral Zr_6_ clusters capped by eight *μ_3_*‐OH ligands. Four of the 12 cluster edges coordinate TCPP‐Fe linkers (5,10,15,20‐tetrakis‐(4‐carboxyphenyl)‐porphyrin‐Fe chloride), while the remaining Zr sites bind 16 terminals ─OH ligands [31, 36, 47]. This architecture confers exceptional POD‐like activity through two synergistic features: (1) iron porphyrin catalytic centers and (2) unsaturated Zr_6_ sites, enabling functionalization. Within MPG(Fe), PEG covalently conjugates MOF‐Fe to GOx, creating a dual‐enzyme system with superior antiparasitic activity compared to either enzyme alone. PEG plays a triple synergistic role in this coupling strategy. First, as a chemical linker, its carboxyl terminus is covalently connected to the unsaturated Zr_6_ site of MOF‐Fe through a solvent‐assisted ligand incorporation method, while its amino terminus is coupled to the carboxyl group of GOx through EDC/NHS chemistry, thereby forming a stable GOx‐PEG‐MOF(Fe) covalent complex. Second, as a flexible spacer arm, it can reduce the spatial steric hindrance between the enzyme and the MOF surface and maintain the catalytic activity of GOx. Finally, as a hydrophilic anti‐fouling layer, it can significantly improve the water solubility and colloidal dispersion of the complex and prevent the particles from aggregating in the electrolyte‐containing biological medium. Stability assays confirmed that covalent PEG conjugation significantly enhances MPG(Fe) robustness over electrostatically adsorbed GOx. This design enables a unique GOx/POD cascade effect, positioning MPG(Fe) as a potent chemotherapeutic agent.

Mechanistically, MOF‐Fe provides POD‐mimetic activity via its iron‐porphyrin centers, while GOx activity was not affected by the structure. Within the intestinal microenvironment, GOx in MPG(Fe) depletes local glucose via conversion to gluconic acid and H_2_O_2_, critically restricting nutrient acquisition by *T. spiralis*. Concomitantly, the generated H_2_O_2_ fuels MPG(Fe)’s POD‐mimetic activity, accelerating •OH radical production, inducing lethal oxidative stress in *T. spiralis*. It is also worth noting that the GOx‐catalyzed production of gluconic acid gradually acidifies the local microenvironment. This acidification may promote parasite killing through two mechanisms. First, it directly disrupts the physiological activities of the intestinal *T. spiralis* that has adapted to the mildly alkaline intestinal environment, while enhancing the POD mimetic activity of the MOF‐Fe component, thereby accelerating the production of •OH. These two effects have a synergistic effect. The reduction of glucose consumption lowers the pH, thereby accelerating the conversion of the co‐generated H_2_O_2_ into cytotoxic •OH. In the body, this acidification is expected to be localized, and most of it will be buffered by the intestinal contents.

The very rapid killing of NBLs is faster than would be expected from oxidative damage alone and warrants consideration of an additional physical component. The granular substances visible in the processed larval images are likely to represent aggregated MPG(Fe) nanoparticles, which may adhere to the surface of the larvae. It is therefore plausible that direct nanoparticle‐membrane contact contributes to membrane rupture and accelerates killing, in addition to the chemical effects. Two observations support a predominantly chemical, concentration‐dependent mechanism rather than a purely mechanical rupture. First, killing was strongly dose‐ and time‐dependent, which is characteristic of a catalytic/oxidative process. Second, MOF‐Fe alone, at the same particle concentrations, exerted no antiparasitic effect, indicating that mere particle contact without the GOx/POD cascade does not kill the worms. Nevertheless, we cannot fully exclude a mechanical contribution, and we have added this as an explicit limitation. The current first‐line treatment drug for trichinellosis is albendazole. Its advantages include being approved for the market, having a low cost, being orally administered, and having certain activity against migrating and encysted muscle larvae. However, it has poor water solubility and low oral bioavailability; and there are risks of liver toxicity, bone marrow suppression, and the possibility of developing drug resistance in veterinary use. MPG(Fe) does not target the microtubule polymerization pathway of the parasite but instead kills intestinal‐stage parasites through a dual, self‐amplifying mechanism, which may avoid cross‐resistance to existing drugs. In terms of biosafety, although no detectable acute toxicity was observed in the mice during the test period, it should be emphasized that the biosafety data presented in this study are solely derived from uninfected healthy mice and the observation period is short. Since *T. spiralis* infection itself induces intestinal barrier disruption and mucosal inflammation, this may alter the absorption, clearance, and toxicity characteristics of MPG(Fe). Therefore, the results of this study cannot be directly extrapolated to infected individuals, and the safety and long‐term toxicity of MPG(Fe) in infected hosts still need to be determined through future research.

## 5. Conclusions

This study provides a proof‐of‐concept that a covalently conjugated GOx/POD‐mimetic cascade nanozyme (MPG[Fe]) can kill intestinal‐stage *T. spiralis* in vitro and markedly reduce adult‐worm burden in a mouse model through the combined actions of glucose starvation and •OH‐mediated oxidative damage. These findings support the further exploration of enzyme‐mimetic nanomaterials as an alternative mechanistic strategy against intestinal helminthiases.

## Author Contributions

**Chuang Zhang:** conceptualization, methodology, chemical experiments, formal analysis, data curation, writing – original draft. **Xiaolei Liu:** investigation, methodology, in vivo experiments, formal analysis, data curation, writing – review and editing, funding acquisition. **Zipei Zhang, Xu Geng, Ning Jiang, and Zhili Hao:** resources, methodology. **Mingyuan Liu and Zhengqiang Li:** writing – review and editing. **Mingyuan Liu:** funding acquisition. **Chen Li:** conceptualization, methodology, formal analysis, data curation, writing – review and editing.

## Funding

This research was funded the Joint Funds of the National Natural Science Foundation of China (Grants U23A20238 and 32230104).

## Ethics Statement

All animal experiments were performed according to regulations of the Administration of Affairs Concerning Experimental Animals in China using protocols that were approved by the Institutional Animal Care and Use Committee of Jilin University (Number SY202511035).

## Consent

The authors have nothing to report.

## Conflicts of Interest

The authors declare no conflicts of interest.

## Supporting Information

Additional supporting information can be found online in the Supporting Information section.

## Supporting information


**Supporting Information** This supporting information supports the main study on the GOx‐MOF cascade complex MPG(Fe) against *Trichinella spiralis*. The morphology, structure, and surface charge of MOF‐Fe, MP(Fe), and MPG(Fe) were confirmed by SEM, TEM, UV–Vis, XRD, and Zeta potential (Figures S1–S4). The complex exhibited pH/temperature‐dependent peroxidase‐like activity, and TA fluorescence, ESR, and Michaelis–Menten analysis directly showed that glucose‐derived H_2_O_2_ is converted to ·OH (Figures S5–S11). Table S1 compares its KM and *V*_max_ with reported peroxidase‐mimicking nanozymes, confirming competitive catalytic efficiency. MPG(Fe) showed good biocompatibility toward IPEC‐J2 cells and significant killing of adult worms and newborn larvae in vitro (Figures S12–S16); in vivo, worm burden decreased markedly with no obvious toxicity (Figures S17–S21). Together, these data confirm that MPG(Fe) combines potent anti‐*Trichinella* activity with favorable biosafety.

## Data Availability

All primary datasets supporting the findings of this study are openly available in the figshare digital repository at https://figshare.com/articles/dataset/Raw_Data/29503964.
