# Supplementary material for: A Natural Enzyme GOx‐MOF Cascade Complex Killing Zoonotic Parasite Trichinella spiralis by Energy Utilization Limitation and Generated Free Radicals
Source: Transbound Emerg Dis. 2026 Sep 18;2026:3527187. doi: 10.1155/tbed/3527187 (PMC13587139; doi:10.1155/tbed/3527187)
Supplement: Supplementary file 1 [file TBED-2026-3527187-s001.docx]

**Supplementary Material**

A natural enzyme GOX-MOF cascade complex killing foodborne parasite *Trichinella spiralis* by energy utilization limitation and generated free radicals

**Figure S1.** a) SEM image of MOF-Fe and MP(Fe). b) TEM image of MOF-Fe and MP(Fe).

**Figure S2.** UV-Vis spectra of TCPP and TCPP-Fe.

**Figure S3.** X-ray diffraction pattern of MOF-Fr, MOF-Fe, MP(Fe) and MPG(Fe).

**Figure S4.** Zeta-Potentials of MOF-Fe, MP(Fe), and MPG(Fe). Bars represent mean ± standard deviation (n = 3).

**Figure S5.** a) Covalent linkage with different ratios (PEG:MOF-Fe) b) Standard curve of PEG concentration measured by Amino acid Concentration Assay Kit (Absorbance at 570nm).

**Figure S6.** a) Standard curve of GOx concentration measured by Bradford protein Concentration Assay Kit; b) The efficiency of GOx loaded by covalent and adsorption under the different pH buffers (100% load defined at pH 7.0).

**Figure S7.** TA was used as a fluorescent probe to detect the formation of ·OH

**Figure S8.** The relative peroxidase-like activity of MPG(Fe) in different pH. a) and temperature b) with the addition of 1mM ABTS, 10 μg mL^-1^ MPG(Fe), 10 mM H_2_O_2_.

**Figure S9.** a) The Michaelis-Menten fitting curve of initial ·OH generation velocities against ABTS concentrations. b) The Lineweaver-Burk fitting (double reciprocal) of the Michaelis-Menten fitting curve.

**Figure S10.** ESR spectra obtained with the addition of 10 μg mL^-1^ MPG(Fe) and 1 mM glucose.

**Figure S11.** a) Standard curve of glucose concentration (DNS method). b) Standard curve of H_2_O_2_ concentration (HRP method). Bars represent mean ± standard deviation (n = 3)

**Figure S12.** Cell viability of IPEC-J2 treated with different MOF-Fe a) and GOx b) for 12h. (N=3) (ns, non-significant; *****p*<0.0001)

**Figure S13.** Live rate of AD in vitro by MOF-Fe a), H_2_O_2_ b), GOx c), (N=3) (*****p*<0.0001, ns, non-significant).

**Figure S14.** Live rate of NBL in vitro by MOF-Fe a), H_2_O_2_ b), GOx c), (N=3)

(***p*<0.01, ****p*<0.001, *****p*<0.0001, ns, non-significant).

**Figure S15.** The fluorescence images of AD incubated with PBS and MPG(Fe) after Hoechst-PI staining for live/death detection.

**Figure S16.** The fluorescence images of NBL incubated with PBS and MPG(Fe) after Hoechst-PI staining for live/death detection.

**Figure S17.** The number with PBS and each treatment group on AD in vivo (N=3) (***p*<0.01, *****p*<0.0001).

**Figure S18.** blood routine examination analyses. WBC (white blood cells), Lymph (lymphocyte), MON (monocytes), Gran (neutrophilic granulocyte), Gran% (neutrophilic granulocyte %), RBC (red blood cells), HGB (hemoglobin), PLT (blood platelet)

**Figure S19.** Statistical analysis of T cells a) and B cells b) for long-term administration of MPG(Fe) with different concentrations. (**p*<0.05, ns, non-significant.

**Figure S20.** Immunofluorescence analysis of splenic tissue T cells and B cells with each treatment group by long-term `administration.

**Figure S21.** Representative images of H&E staining of the heart, liver, spleen, lung and kidney with each treatment group by long-term administration (N=3).

**Table S1** Comparison of POD-like activity in apparent Michaelis-Menten constant (KM) and maximum reaction rate (Vmax) between MPG(Fe) and Reported POD-like nanozyme.

|  | Substrate | *K_M_*（mM） | *V*_max_（10^-8^M·s^-1^） | Ref. |
| --- | --- | --- | --- | --- |
| MPG（Fe） | ABTS | 0.031 | 7.205 | This work |
|  | H_2_O_2_ | 5.81 | 7.223 |  |
| Hemin@MOF | H_2_O_2_ | 10.9 | 8.98 | Qin, et al.,2013 |
| CeO_2_ | H_2_O_2_ | 64.6 | 5.07 | Jiao, et al.,2012 |
| AuNPs@g-C3N4 | H_2_O_2_ | 12.3 | 9.0 | Wu, et al.,2019 |
| Fe_3_O_4_ | H_2_O_2_ | 154 | 9.78 | Gao, et al.,2007 |
| ILE incorporated nanoflowers | H_2_O_2_ | 1.02 | 5.77 | Jiang, et al.,2021 |
| HRP | H_2_O_2_ | 3.70 | 8.71 | Jiang, et al.,2021 |

**Supplementary Description**

This supplementary material supports the main study on the GOx-MOF cascade complex MPG(Fe) against Trichinella spiralis. The morphology, structure, and surface charge of MOF-Fe, MP(Fe), and MPG(Fe) were confirmed by SEM, TEM, UV-Vis, XRD, and Zeta potential (Figures S1–S4). The complex exhibited pH/temperature-dependent peroxidase-like activity, and TA fluorescence, ESR, and Michaelis-Menten analysis directly showed that glucose-derived H₂O₂ is converted to ·OH (Figures S5–S11). Table S1 compares its KM and Vmax with reported peroxidase-mimicking nanozymes, confirming competitive catalytic efficiency. MPG(Fe) showed good biocompatibility toward IPEC-J2 cells and significant killing of adult worms and newborn larvae in vitro (Figures S12–S16); in vivo, worm burden decreased markedly with no obvious toxicity (Figures S17–S21). Together, these data confirm that MPG(Fe) combines potent anti-Trichinella activity with favorable biosafety.

**Reference**

1.	Qin F-X, Jia S-Y, Wang F-F, Wu S-H, Song J, Liu Y. Hemin@ metal–organic framework with peroxidase-like activity and its application to glucose detection. Catalysis Science & Technology. 2013;3(10):2761-8.

2.	Jiao X, Song HJ, Zhao HH, Bai W, Zhang LC, Lv Y. Well-redispersed ceria nanoparticles: Promising peroxidase mimetics for H2O2 and glucose detection. Anal Methods-Uk. 2012;4(10):3261-7.

3.	Wu N, Wang YT, Wang XY, Guo FN, Wen H, Yang T, et al. Enhanced peroxidase-like activity of AuNPs loaded graphitic carbon nitride nanosheets for colorimetric biosensing. Anal Chim Acta. 2019;1091:69-75.

4.	Gao L, Zhuang J, Nie L, Zhang J, Zhang Y, Gu N, et al. Intrinsic peroxidase-like activity of ferromagnetic nanoparticles. Nat Nanotechnol. 2007;2(9):577-83.

5.	Jiang N, Zhang C, Li M, Li S, Hao Z, Li Z, et al. The Fabrication of Amino Acid Incorporated Nanoflowers with Intrinsic Peroxidase-like Activity and Its Application for Efficiently Determining Glutathione with TMB Radical Cation as Indicator. Micromachines (Basel). 2021;12(9).
